# The role of indoxyl sulfate in renal anemia in patients with chronic kidney disease

**DOI:** 10.18632/oncotarget.18789

**Published:** 2017-06-28

**Authors:** Chih-Jen Wu, Cheng-Yi Chen, Thung-S. Lai, Pei-Chen Wu, Chih-Kuang Chuang, Fang-Ju Sun, Hsuan-Liang Liu, Han-Hsiang Chen, Hung-I. Yeh, Chih-Sheng Lin, Cheng-Jui Lin

**Affiliations:** ^1^ Division of Nephrology, Department of Internal Medicine, Mackay Memorial Hospital, Mackay Medical College ,Taipei, Taiwan; ^2^ Department of Medical Research, China Medical University Hospital, China Medical University, Taichung, Taiwan; ^3^ Graduate Institute of Medical Sciences and Department of Pharmacology, School of Medicine, College of Medicine, Taipei Medical University, Taipei, Taiwan; ^4^ Graduate Institute of Biomedical Science, Mackay Medical College, New Taipei City, Taiwan; ^5^ Institute of Biotechnology, National Taipei University of Technology, Taipei, Taiwan; ^6^ Division of Genetics and Metabolism, Department of Medical Research, Mackay Memorial Hospital, Taipei, Taiwan; ^7^ College of Medicine, Fu-Jen Catholic University, Taipei, Taiwan; ^8^ Mackay Junior College of Medicine, Nursing and Management, Taipei, Taiwan; ^9^ Department of Medical Research, Mackay Memorial Hospital, Taipei, Taiwan; ^10^ Department of Medicine, Mackay Medical College, New Taipei City, Taiwan; ^11^ Department of Biological Science and Technology, National Chaio Tung University, Hsinchu, Taiwan

**Keywords:** indoxyl sulfate, chronic kidney disease, erythropoietin, renal anemia

## Abstract

Renal anemia is a common complication in patients with advanced chronic kidney disease. *In vitro* studies have shown that indoxyl sulfate decreases erythropoietin production. Whether this effect is seen *in vivo* remains unclear. Our goal was to explore the role of indoxyl sulfate in renal anemia. We found serum indoxyl sulfate levels are significantly and negatively associated with erythropoietin levels in human. A multiple stepwise linear regression analyses after adjustment for other independent parameters revealed that free indoxyl sulfate, and total indoxyl sulfate were significantly associated with erythropoietin levels. In animal studies, erythropoietin gene and protein expression were markedly inhibited in rats with chronic kidney disease; however, this effect was significantly reversed by lowering serum indoxyl sulfate with AST-120. Indoxyl sulfate may also inhibit erythropoietin expression in animal models with chronic kidney disease. These findings further support the role of indoxyl sulfate in the development of renal anemia.

## INTRODUCTION

Anemia is a well-known consequence of chronic kidney disease (CKD), and the third National Health and Nutrition Examination Survey has shown that the prevalence of anemia increases in subjects with an estimated glomerular filtration rate (eGFR) of less than 60 mL/min/1.73 m^2^ [1]. The lower the hematocrit, the greater the risk of developing ESRD [2]. Hypoxia of the tubular cells plays an important role in tubulointerstitial damage associated with CKD, and in the progression of renal failure [3]. Three randomized studies have suggested that anemia correction may slow the progression of CKD [4-6]. Anemia commonly contributes to a poor quality of life, and also significantly reduces patient survival.

Patients with advanced CKD encounter anemia due to insufficient erythropoietin (EPO) production by the peritubular fibroblasts of the diseased kidney [7]. Expression of EPO is controlled by hypoxia-inducible transcription factor (HIF), which is a heterodimeric transcription factor composed of a hypoxia-inducible alpha-subunit, and a constitutively expressed beta-subunit. Indoxyl sulfate (IS), a colon derived uremic solute, has been reported to contribute to the development of uremic symptoms [8, 9]. Recent studies have indicated that serum IS level is a valuable marker for predicting atherosclerosis, cardiovascular disease, renal function decline, and peripheral arterial disease in patients with advanced chronic kidney disease [10-14]. Furthermore, IS may inhibit the HIF system in addition to aggravating the hypoxia of the kidney, by causing functional impairment of the HIF-1-alpha C-terminal transactivation domain [15]. As revealed by a cell and animal study, IS was able to impair oxygen-sensing in EPO-producing cells, suppressing renal EPO messenger RNA expression and the plasma EPO levels [16]. These results showed the toxic effects of IS on EPO synthesis in vitro. However, whether this effect can be seen in humans remains unclear. The aim of our study was to investigate the role of IS in renal anemia in a CKD cohort and animal model. The results of our study reveal the relationship between IS and EPO levels.

## RESULTS

### Patient characteristics

One hundred and thirteen stable patients with CKD were recruited in the study, and the demographic and clinical characteristics of the patients are given in Table 1 . This study population consisted of 57 males (50.4%) and 56 females (49.6%), with a mean age of 57.1 ± 14.4 years. Fifty-six patients had diabetes mellitus (49.6%), 55 patients had hypertension (48.7%), and 48 patients had cardiovascular disease (42.8%). Among them, 17 patients (15%) were classified as CKD stage-2, 27 patients (23.8%) were classified as CKD stage-3, 41 patients (36.2%) were classified as CKD stage-4, and 28 patients (24.8%) were classified as CKD stage-5. The clinical parameters were as follows: albumin: 4.5 ± 0.5 (g/dL); hematocrit: 26.8 ± 6.0 (%); blood urine nitrogen: 57.3 ± 22.5 (mg/dL); creatinine: 3.1 ± 2.4 (mg/dL); estimated GFR: 20.9 ± 23.7 (ml/min/1.73m^2^); calcium: 9.1 ±0.8 (mg/dL); phosphate: 4.3 ± 1.1 (mg/dL); c-reactive protein: 0.5 ± 0.3 (mg/dL); ferrous: 64.3 ± 23.7 (μg/dL), ferritin: 196.9 ± 95.2 (ng/mL) and EPO: 12.2 ±11.9 (mIU/ml). The total IS (T-IS) and free IS (F-IS) were 15.8 ± 6.9 and 0.22 ± 0.6 mg/L, respectively.

**Table 1:** Baseline characteristics of the study patients.

Variables	All (n=113)
Age (yr)	57.1 ± 14.4
Male (%)	50.4
Diabetes Mellitus (%)	49.6
Hypertension (%)	48.7
Cardiovascular disease (%)	42.8
SBP (mmHg)	144.6 ± 16.1
DBP (mmHg)	75.7 ± 10.5
CKD stage (%)	
2	15.2
3	23.8
4	36.2
5	24.8
Albumin (g/dL)	4.5 ± 0.5
Hematocrit (%)	26.8 ± 6.0
BUN (mg/dL)	57.3 ± 22.5
Creatinine (mg/dL)	3.1 ± 2.4
eGFR (ml/min/1.73m^2^)	20.9 ± 23.7
Calcium (mg/dL)	9.1 ± 0.8
Phosphate (mg/dL)	4.3 ± 1.1
CRP (mg/dL)	0.51 ± 0.32
Fe (ug/dL)	64.3 ± 23.7
Ferritin (ng/mL)	196.9 ± 95.2
Free IS (mg/L)	0.2 ± 0.6
Total IS (mg/L)	15.8 ± 6.9
EPO (mIU/ml)	12.2 ± 11.9

PS: values were expressed as mean ± SD unless otherwise defined.

Abbreviation: BUN: blood Urea Nitrogen; eGFR: estimated glomerular filtration rate; CRP: C-reactive protein; Fe: ferrous; IS: indoxyl sulfate.; EPO: erythropoietin.

### Correlation between EPO and the clinical variables

**Figure 1:**
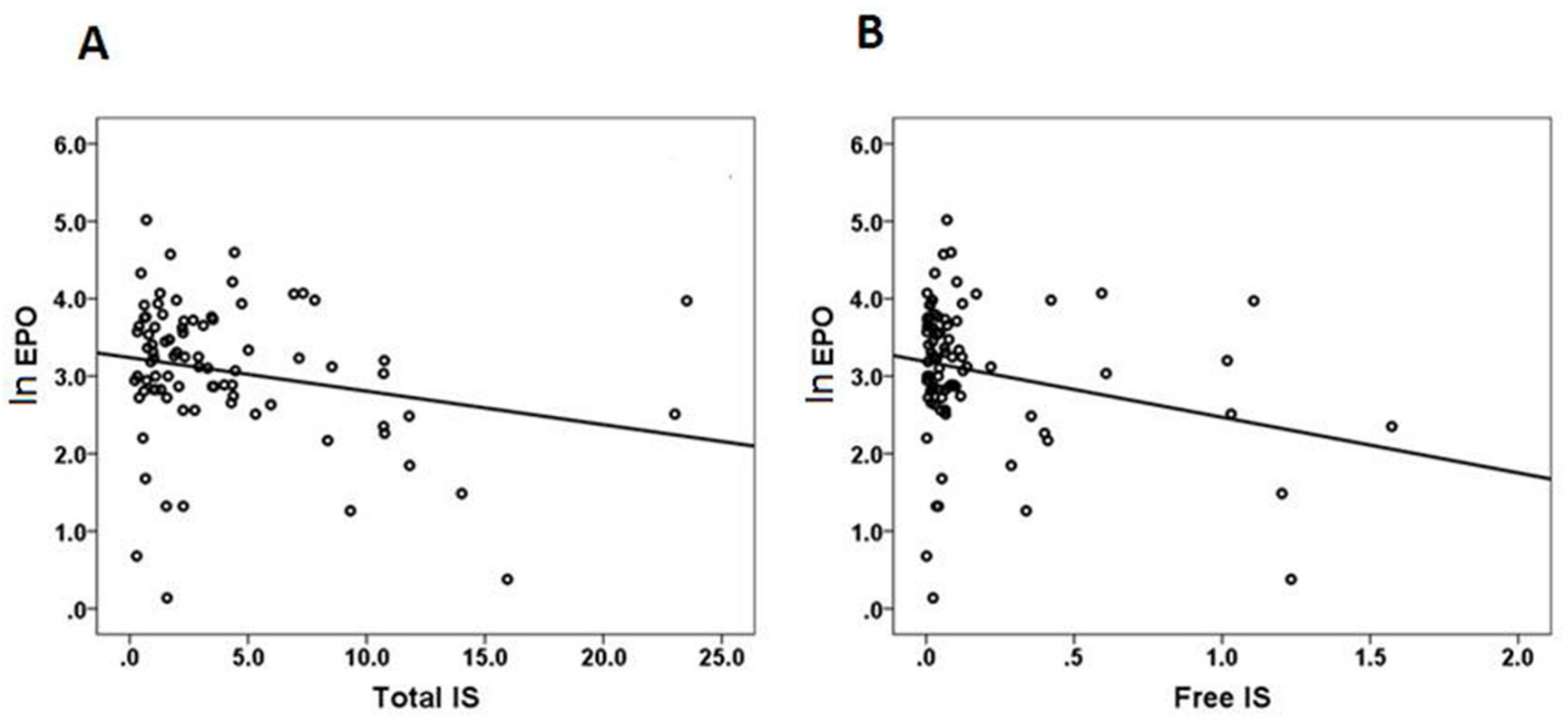
Agreement between EPO and IS analyzed by Pearson’s correlation **(A)** ln EPO vs T-IS, r=-0.22, p=0.041 **(B)** ln EPO vs F-IS, r=-0.25, p=0.021.

The results of the simple and multiple stepwise linear regression analyses for EPO as a dependent variable are shown in Table 2 . On the simple linear regression analyses, the EPO was significantly correlated to hematocrit (B=0.02, *P*=0.032), free IS (B=-0.50, *P*=0.021), and total IS (B=-0.03, *P*=0.041). Because of collinearity between free and total IS, we used hematocrit and free IS in model I, and hematocrit and total IS in model II. After adjusting for other independent parameters, only serum free IS (B=-0.55, *p*=0.008) was significantly associated with EPO levels on multiple variable analysis (model I). There was also a significantly negative correlation between EPO and total IS (B=-0.04, *p*=0.013) in model II. Moreover, the agreement between EPO and total IS was analyzed by Pearson’s correlation. EPO levels were significantly correlated with total IS (*r*=-0.22, *p*=0.041) (Figure 1A) and free IS (*r*=-0.25, *p*=0.021)( Figure 1B ).

**Table 2:** Simple and multiple stepwise linear regression analyses for EPO as a dependent.

	Simple variable analysis			Multiple variables analysis(Model I)			Multiple variables analysis(Model II)		
	B	95% C.I.	*p*	B	95% C.I.	*p*	B	95% C.I.	*p*
Sex(male/female)	0.05	-0.22∼0.33	0.693						
Age(y)	-0.01	-0.01∼0.01	0.915						
DM (yes/no)	-0.16	-0.44∼0.12	0.252						
Hypertension(yes/no)	0.04	-0.23∼0.31	0.771						
CVD(yes/no)	0.29	-0.07∼0.65	0.114						
CKD stages	-0.10	-0.22∼0.01	0.086						
Hematocrit(%)	0.02	0.00∼0.05	0.032						
Albumin (g/dL)	0.02	-0.33∼0.38	0.906						
BUN(mg/dL)	-0.01	-0.01∼0.00	0.227						
Creatinine(mg/dL)	-0.05	-0.12∼0.02	0.132						
eGFR(ml/min/1.73m^2^)	0.01	0.00∼0.01	0.409						
Sodium (mg/dL)	0.03	-0.02∼0.08	0.280						
Potassium(mg/dL)	-0.12	-0.37∼0.13	0.335						
Calcium(mg/dL)	0.06	-0.14∼0.25	0.553						
Phosphate(mg/dL)	-0.08	-0.24∼0.09	0.349						
CRP(mg/dL)	0.03	-0.50∼0.56	0.904						
Fe(ug/dL)	-0.01	-0.01∼0.01	0.727						
Ferritin(ng/mL)	-0.01	0.00∼0.00	0.879						
Free IS(mg/L)	-0.50	-0.92∼-0.08	0.021	-0.55	-0.96∼-0.15	0.008			
Total IS(mg/L)	-0.03	-0.06∼0.00	0.041				-0.04	-0.06∼-0.01	0.013

### Association between IS and EPO expression status in the animal study

**Figure 2:**
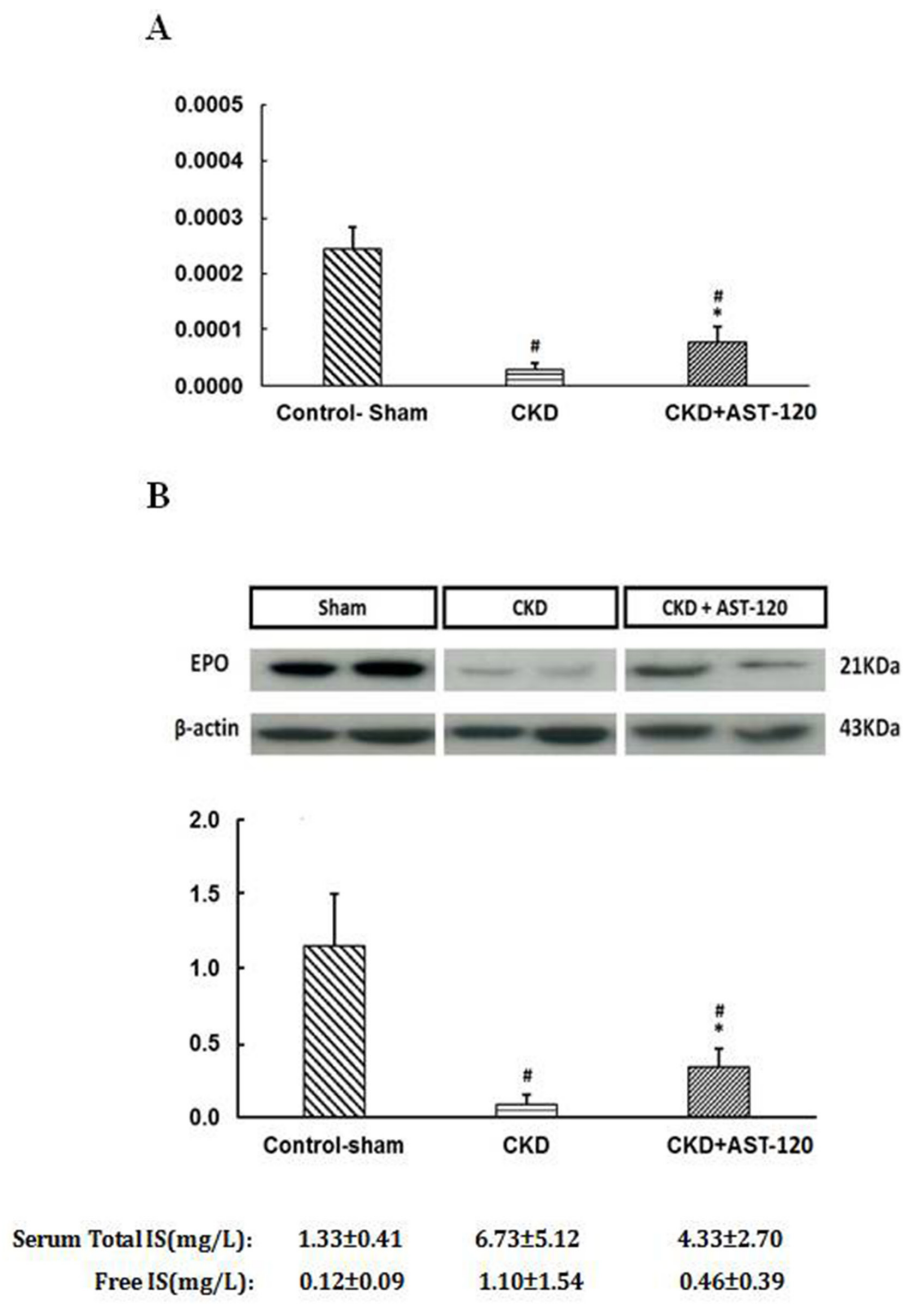
The EPO expression status in an animal model **(A)** Real-time quantitative PCR was performed to assess the change of EPO mRNA expression in control-sham, CKD and CKD+AST-120 group. EPO mRNA was suppressed in CKD rat. It was restored in rats with feeding AST-120 by lowering serum IS. **(B)** Western blot analysis. EPO protein expression was also decreased significantly in CKD rat. AST-120 could reverse the inhibition of EPO expression in CKD. n=5 for each, #, p<0.01 compared to control-sham group, *, p<0.05 compared CKD group.

All animals were divided into three groups: control-sham, CKD, and CKD + AST-120 groups. The serum total and free IS levels were increased in CKD group (6.73 ± 5.12, 1.10 ± 1.54 mg/L, respectively), and were lowered when treated with AST-120 (4.33 ± 2.70, 0.46 ± 0.39 mg/L, respectively). In Figure 2A , the EPO mRNA expression was inhibited in CKD rats versus the control group (*P*<0.01), and significantly increased when treated with AST-120 versus the CKD group, measured by quantitative RT-PCR (*P*<0.05). However, the EPO protein expression analyzed using the western blotting method was also decreased in CKD group, and this inhibition in CKD group was reversible in CKD + AST-120 group (*P*<0.05) (Figure 2B). These data indirectly demonstrated that IS may be involved in the development of renal anemia.

## DISCUSSION

The novel finding of the present study is that total IS has been significantly associated with the EPO levels in a CKD cohort. Moreover, the inhibition of EPO expression by IS has also been indirectly confirmed on the animal model. These findings further support the role of IS in the regulation and development of renal anemia in CKD patients.

The main cause of renal anemia in advanced CKD is a deficient production of EPO. According to the study reported by Bernhardt, et al., the pharmacological activation of hypoxia-inducible transcription factors (HIFs) in dialysis patients increases the EPO production, suggesting that the desensitization of the oxygen-sensing mechanism is responsible for the development of renal anemia [17]. The HIF is a master transcriptional regulator of the adaptive responses against hypoxia, and regulates the expression of EPO, angiogenic factors, glycolytic enzymes, glucose transporters, anti-oxidative enzymes, and other protective mechanisms [18].

IS is protein-bound uremic toxin derived from the fermentation of tryptophan by the intestinal flora. Evidence suggests that the kidney, liver function and gut determine the serum IS levels [12, 19, 20]. Moreover, IS may be involved in the regulation of renal anemia. Chiang et al. provided evidence that links IS to the impairment of EPO production through a reduced nuclear accumulation of HIFs following hypoxia in HepG2 cells [16]. The finding that the administration of indole leads to the suppression of renal EPO mRNA expression and decreases plasma EPO levels, is also confirmed on the animal model. Another report suggest that IS could suppress EPO expression in an HIF-independent manner, by inducing endoplasmic reticulum stress and thereby contributing to the progression of cellular damage in tubular epithelial cells [21]. Moreover, IS also suppresses the EPO receptor Akt signaling pathway in the extra-hematopoietic cells, leading to EPO resistance in the human umbilical vein endothelial cells [22]. Another *in vitro* study has reported that IS stimulates suicidal erythrocyte death (eryptosis), an effect in large part due to extracellular Ca^2+^ entry with subsequent stimulation of cell shrinkage and cell membrane scrambling [23].

These basic studies suggest that IS plays a role in the development of renal anemia.

However, there is little direct evidence to demonstrate the relationship between IS levels and the EPO concentration in humans. One previous clinical study has revealed that in hemodialysis patients the IS levels are increased and the patients require a higher dosage of EPO [24]. Another randomized, fixed dose, controlled study has reported that in advanced CKD patients the adjuvant administration of AST-120 (an active charcoal that can effectively absorb IS) is associated with an improvement in the renal function and hemoglobin levels, versus the use of the EPO stimulating agent alone [25]. This suggests that using AST-120 improves renal anemia not only by reducing IS, but also by improving the EPO production. These two studies have provided indirect evidence linking the correlation of IS and EPO levels.

To our knowledge, our study is the first to demonstrate the relationship between IS and the EPO concentration in humans, after adjusting for other confounding variables. Our results provide a direct evidence of the association of IS and the EPO levels in CKD patients. Moreover, our animal model also confirmed that lowering the uremic toxin can increase the EPO expression in CKD rat when treated with AST-120. It indirectly reflects that IS may play an inhibitory role in the EPO production. The inadequate synthesis of EPO can also be reversed by reducing IS with AST-120.

One study published by Palm, et al. has reported that IS dysregulates the oxygen metabolism and enhances the oxygen consumption in the tubular cells, thus aggravating tubule interstitial hypoxia, which can be reversed by AST-120 in the remnant kidney [26]. This suggests that AST-120 may help in preserving more renal EPO-producing cells, enhancing the EPO activity, and ameliorating the EPO resistance, by absorbing IS. These evidences reinforce the association between the uremic status and inadequate synthesis of EPO. Thus, these findings might have positive impact on the future CKD management. In addition to adequate dose of EPO injection, how to lower serum IS is also an essential step to synergistically improve renal anemia in patients with advanced CKD.

There are some limitations of our study. First, the number of patients is small, as only patients from one regional hospital were included in the study. Second, the association between IS and the nuclear fraction of HIF-1-alpha after AST-120 administration was not confirmed in this study. Third, our study is a cross-sectional design, so we cannot answer the questions about the definite mechanism and interactions between IS and the EPO levels. Fourth, we did not measure p-cresyl sulfate (PCS), another protein-bound uremic solute in this study. Although, both IS and PCS exhibit a similar effect on clinical outcomes [27–29], there are some differences between them. There is also little evidence from *in vitro* studies indicating that PCS dysregulates HIFs. It is unclear if PCS is also involved in the regulation of renal anemia. Thus, further prospective investigations are required to clarify this intriguing question.

In conclusion, our study reveals that the serum EPO levels are significantly and negatively associated with serum IS in CKD patients. Our animal study has also indirectly demonstrated the association between IS and the EPO expression. These findings further support the idea that IS is a key factor in renal anemia and the regulation of the EPO production in CKD.

## MATERIALS AND METHODS

### Subjects

This study recruited 113 patients with stable CKD (stage 2, 3, 4 and 5) from our institution’s outpatient department. Patients with acute infection, liver cirrhosis, malignancy, or those who were younger than 18 years of age were excluded from the study. The etiology of CKD among the patients included type-2 diabetic nephropathy (49.6%), and chronic glomerular nephritis (50.4%). The study included human and animal research, and was performed according to the principles of the Declaration of Helsinki, and approved by the ethics committee of the Mackay Memorial Hospital in Taiwan. All patients gave written Informed consent before the study was initiated.

### Laboratory assessment

After enrollment, fasting blood samples were obtained from all patients with CKD. The estimated glomerular filtration rate (eGFR) was calculated using the Modification of Diet in Renal Disease equation as follows: 175 × (S_cr_)^-1.154^ × (Age)^-0.203^ × (0.742 if female). The following data were collected: blood urea nitrogen (BUN)(mg/dL), creatinine (mg/dL), hemoglobin (g/dL), hematocrit (%), calcium (Ca), (mg/dL), phosphate (mg/dL), albumin (g/dL), IS (mg/L) and EPO (μIU/mL). EPO was measured with a human ELISA Kit (R&D systems, USA). The bromocresol green method was used to determine the serum albumin levels.

### Measurement of IS

Serum IS levels were analyzed using LC-MS/MS (4000 QTRAP, USA). Briefly, serum samples were prepared and deproteinized by heat denaturation. The free concentrations of IS were measured in serum ultrafiltrates, which were obtained using Microcon YM-30 separators (Millipore, Billerica, MA, USA), followed by the same sample preparation and analysis methods used for analyzing the serum levels of IS: High Performance Liquid Chromatography (HPLC) was performed at room temperature using a dC18 column (3.0 × 50 mm, Atlantis, Waters). The buffers used were (A) 0.1% formic acid and (B) 1 mM NH_4_OAc plus 0.1% formic acid in 100% acetonitrile. The flow rate was 0.6 mL/min with a 3.5-min gradient cycling from 90% A/10% B to 10% A/90% B. Under these conditions, IS was eluted at 2.48 minutes. Standard curves for IS were set at 1, 5, 10, 50, 250, 500 and 1,000 μg/L, and correlated with the serum samples, with average r^2^ values of 0.996 ± 0.003. These samples were diluted if the IS concentration exceeded a standard curve. Quantitative results were obtained and calculated in terms of their concentrations (mg/L). The sensitivity of this assay was 1 μg/L for IS.

### Animals

#### CKD rat (5/6 nephrectomy)

Six-week-old male Sprague-Dawley rats (weight 160–180 g) were purchased from Lasco Taiwan Inc. At 8 weeks of age (weight 210–230 g), the rats were anesthetized with Zoletil 50/Xylazine (6 mg/1.3 mg/kg body weight), and the upper and lower thirds of the right kidney were removed. One week after the first operation, the left kidney was removed, leaving approximately 1/6 of the total renal mass. Sham operated rats were used as controls. The animals had free access to tap water and standard chow. Two weeks after the 5/6 nephrectomy, the rats were randomized to receive either AST-120 (5/6-nephrectomy + AST-120) or no treatment (5/6-nephrectomy) for 4-weeks. The AST-120 (Kremezin, Kureha Pharmaceuticals, Tokyo, Japan) was administrated post-operatively in the chow at 8% w/w. The remnant kidneys were removed after perfusion at the end of experiment, for EPO gene and protein expression studies. All animal were divided into three groups: control-sham, CKD, and CKD + AST120 groups.

The local committee for care and use of laboratory animals at the Mackay Memorial Hospital approved this study. In this study, 3 of 18 rats died after surgery, while the other rats survived until the end of study. All experimental procedures of animal were performed according to Institutional Animal Care and Use Committee (IACUC) policy. All rats were monitored every day and were treated with meperidine (0.3-0.5 mg/100g body weight for pain control (if need) based on the physiological parameters as following: 1. Avoidance, vocalization and aggressiveness (mainly if the animal cannot escape). 2. Spontaneous activities are reduced. The animal is isolated from the social group. 3. Altered gait. 4. Hunched posture. 5. Pilo-erection. 6. Reduced grooming; dark-red stain around the eyes and at nostrils. 7. Reduced appetite and subsequent weight loss. 8. Increased respiration rate. 9. Failure to explore cage when disturbed. We followed IACUC policy by using CO_2_ Euthanasia as the method of sacrifice at the end of study.

#### Quantitative reverse transcriptase-polymerase chain reaction (RT-PCR)

Total RNA was extracted from the renal cortex trimmed from fresh frozen renal tissue using the Trizol Reagent (Life Technologies), as per the manufacturer recommended protocol. The renal cortex (150 mg) was homogenized with 1 mL Trizol, and the RNA-containing aqueous phase was obtained by centrifugation. Following the precipitation of RNA by centrifugation with 0.5 volume isopropanol and purification with 75% ethanol, the extracted total RNA was dissolved in 80 μL nuclease-free deionized distilled water, and stored at -20°C.The concentration and purity of the RNA were determined by using the Nanodrop Spectrophotometer (Thermo Scientific). First-strand cDNA was synthesized from the template RNA (1 μg) using Transcriptor First Strand cDNA Synthesis Kit (Roche), according to the manufacturer recommended protocol. A real-time PCR was performed using a 2 μL template in a 20 μL reaction, containing 1 μM of each primer and 10 μL of Light Cycler Fast Start Universal SYBR Green Master (ROX) (Roche).

The following Lightcycler^®^96 conditions were used: Pre-incubation at 95°C for 10 minutes, followed by 40 cycles with 2-step amplification at 95°C for 10 seconds, 60°C for 30 seconds, melting at 95°C for 10 seconds, 65°C for 60 seconds, 97°C for 1 second, and cooling at 37°C for 30 seconds (Roche). Glyceraldehyde 3-phosphate dehydrogenase (GAPDH) was used as a housekeeping gene, to normalize the gene expression data.

The primer sequences were as follows: TACGTAGCCTCACTTCACTGCTT and GCAGAAAGTATCCGCTGTGAGTGTTC. The PCR was conducted in triplicate for each sample.

### Western blotting analysis

For determining the EPO protein, 30 μg nuclear extract protein was loaded per lane.

These protein samples were separated by electrophoresis on an 8% SDS-polyacrylamide gel, followed by an electro-transfer to polyvinylidene difluoride membranes (GE Healthcare Bio-Sciences, Little Chalfont, UK). The transferred membranes were blocked with 5% fat-free skim milk in tris-buffered saline, with 0.01% Tween 20 for 60 minutes at room temperature. The membrane was incubated with rabbit polyclonal anti-EPO antibody (diluted 1:1000 in PBS/5% skim milk; Novus. Biologicals), overnight at 4°C. Horseradish peroxidase (HRP) conjugated anti-rabbit IgG (Promega) was then used as the secondary antibody. The immunoreactive protein was visualized by using the chemiluminescent protocol (ECL, GE HealthcareBio-Sciences). Anti-actin rabbit polyclonal antibody (1:1000; Sigma-Aldrich) was used for the calibration. We employed beta-actin as an internal control.

### Statistical analysis

The human demographic data were expressed as the mean ± SD. Independent clinical variables were ln transformed, if not normally distributed. The agreement between EPO and IS were analyzed by Pearson’s correlation. To assess the influence of the tested parameters, the simple and multiple stepwise linear regression analyses (we put all significant variables in a univariate linear regression analysis) were used to evaluate EPO as a dependent variable. In animal studies, data were expressed as mean ± SEM. One-way ANOVA with Tukey’s post hoc test was used to compare the differences between the control and study groups in the animal model. A *P* value of less than 0.05 was considered to be statistically significant. All statistical analyses were conducted using the SPSS ver. 21.0 software program (IBM, Armonk, New York, USA).
