# Correction: The role of indoxyl sulfate in renal anemia in patients with chronic kidney disease

**DOI:** 10.18632/oncotarget.26782

**Published:** 2019-03-08

**Authors:** Chih-Jen Wu, Cheng-Yi Chen, Thung-S. Lai, Pei-Chen Wu, Chih-Kuang Chuang, Fang-Ju Sun, Hsuan-Liang Liu, Han-Hsiang Chen, Hung-I. Yeh, Chih-Sheng Lin, Cheng-Jui Lin

**Affiliations:** ^1^ Division of Nephrology, Department of Internal Medicine, Mackay Memorial Hospital, Mackay Medical College, Taipei, Taiwan; ^2^ Department of Medical Research, China Medical University Hospital, China Medical University, Taichung, Taiwan; ^3^ Graduate Institute of Medical Sciences and Department of Pharmacology, School of Medicine, College of Medicine, Taipei Medical University, Taipei, Taiwan; ^4^ Graduate Institute of Biomedical Science, Mackay Medical College, New Taipei City, Taiwan; ^5^ Institute of Biotechnology, National Taipei University of Technology, Taipei, Taiwan; ^6^ Division of Genetics and Metabolism, Department of Medical Research, Mackay Memorial Hospital, Taipei, Taiwan; ^7^ College of Medicine, Fu-Jen Catholic University, Taipei, Taiwan; ^8^ Mackay Junior College of Medicine, Nursing and Management, Taipei, Taiwan; ^9^ Department of Medical Research, Mackay Memorial Hospital, Taipei, Taiwan; ^10^ Department of Medicine, Mackay Medical College, New Taipei City, Taiwan; ^11^ Department of Biological Science and Technology, National Chiao Tung University, Hsinchu, Taiwan

**This article has been corrected:** The correct affiliation information is given below:

^11^ Department of Biological Science and Technology, National Chiao Tung University, Hsinchu, Taiwan

Original article: Oncotarget. 2017; 8:83030-83037. https://doi.org/10.18632/oncotarget.18789

